# Interleukin-4 and interleukin-13 induce different metabolic profiles in microglia and macrophages that relate with divergent outcomes after spinal cord injury

**DOI:** 10.7150/thno.65203

**Published:** 2021-10-03

**Authors:** Jesus Amo-Aparicio, Joana Garcia-Garcia, Isaac Francos-Quijorna, Andrea Urpi, Anna Esteve-Codina, Marta Gut, Albert Quintana, Ruben Lopez-Vales

**Affiliations:** 1 Departament de Biologia Cellular, Fisiologia i Immunologia, Institut de Neurociències, Universitat Autònoma de Barcelona, Bellaterra, 08193, Catalonia, Spain.; 2 Regeneration Group, Wolfson Centre for Age-Related Diseases, IoPPN, King's College London, London, SE11YR, UK.; 3 CNAG-CRG, Centre for Genomic Regulation (CRG), Barcelona Institute of Science and Technology (BIST), Baldiri i Reixac 4, Barcelona, 08028, Catalonia, Spain.; 4 Universitat Pompeu Fabra (UPF), Barcelona, 08020, Catalonia, Spain.; 5 Centro de Investigación Biomédica en Red sobre Enfermedades Neurodegenerativas (CIBERNED).

**Keywords:** interleukin 4, interleukin 13, immune metabolism, polarization, spinal cord injury

## Abstract

**Background:** Microglia and macrophages adopt a pro-inflammatory phenotype after spinal cord injury (SCI), what is thought to contribute to secondary tissue degeneration. We previously reported that this is due, in part, to the low levels of anti-inflammatory cytokines, such as IL-4. Since IL-13 and IL-4 share receptors and both cytokines drive microglia and macrophages towards an anti-inflammatory phenotype *in vitro*, here we studied whether administration of IL-13 and IL-4 after SCI leads to beneficial effects.

**Methods:** We injected mice with recombinant IL-13 or IL-4 at 48 h after SCI and assessed their effects on microglia and macrophage phenotype and functional outcomes. We also performed RNA sequencing analysis of macrophages and microglia sorted from the injured spinal cords of mice treated with IL-13 or IL-4 and evaluated the metabolic state of these cells by using Seahorse technology.

**Results:** We observed that IL-13 induced the expression of anti-inflammatory markers in microglia and macrophages after SCI but, in contrast to IL-4, it failed to mediate functional recovery. We found that these two cytokines induced different gene signatures in microglia and macrophages after SCI and that IL-4, in contrast to IL-13, shifted microglia and macrophage metabolism from glycolytic to oxidative phosphorylation. These findings were further confirmed by measuring the metabolic profile of these cells. Importantly, we also revealed that macrophages stimulated with IL-4 or IL-13 are not deleterious to neurons, but they become cytotoxic when oxidative metabolism is blocked. This suggests that the metabolic shift, from glycolysis to oxidative phosphorylation, is required to minimize the cytotoxic responses of microglia and macrophages.

**Conclusions:** These results reveal that the metabolic fitness of microglia and macrophages after SCI contributes to secondary damage and that strategies aimed at boosting oxidative phosphorylation might be a novel approach to minimize the deleterious actions of microglia and macrophages in neurotrauma.

## Introduction

Spinal cord injury (SCI) results in loss of motor, sensory, and autonomic functions 1. SCI triggers two phases of neurodegeneration after SCI known as primary and secondary injury. While the first refers to the damage induced by the initial trauma itself in the spinal cord 1, secondary injury includes a sequence degenerative events that take place in the spinal cord during the following days or weeks 2. Among the different events that participate in secondary damage, inflammation is one of the main contributors 3,4.

The inflammatory response elicited in the spinal cord parenchyma after lesion is orchestrated mainly by tissue-resident microglia and blood-infiltrated macrophages 5. These two types of cells participate in the clearance of the cellular and myelin debris from the injury site, in part, by secreting cytotoxic factors such as free radical species, complement proteins, and proteases, among other molecules 3. However, as occurs with most of the functions elicited by innate immunity, these cytotoxic factors are unspecific and affect healthy neighboring tissue leading to cell death, increased lesion size, and functional impairments 6. Indeed, treatment with several anti-inflammatory drugs reduces tissue damage and functional deficits after SCI 7,8. However, clearance of cellular and myelin debris, together with the release of some helpful factors by microglia and macrophages, are critical for wound healing and axon regeneration 3. In fact, approaches aimed at attenuating the infiltration of monocytes or the expansion of microglia resulted into detrimental effects 9,10.

Microglia and macrophages can therefore mediate beneficial and harmful responses after SCI. The mechanisms that influence the outcome of the two immune cells are now beginning to be understood. Extensive *in vitro* studies have revealed that stimulation of microglia or macrophages with pro-inflammatory factors such as LPS or INFγ leads to a pro-inflammatory and cytotoxic state (also known as M1), whereas stimulation with cytokines such as IL-4 or IL-13 results in anti-inflammatory and pro-repair phenotype (also known as M2) 11,12. Although this binary classification of microglia and macrophages is useful when performing* in vitro* studies, these cells are exposed to multiple factors *in vivo* cells, with pro-inflammatory and anti-inflammatory actions, whose concentrations may vary at any given time. This results in a wide spectrum of phenotypes that can be more skewed towards pro-inflammatory or anti-inflammatory phenotype 13. In the present study, the terms detrimental/pro-inflammatory and protective/anti-inflammatory will be used to refer to the polarization states of macrophages and microglia bearing in mind that they are just an experimental construct.

Interleukin-13 (IL-13) is one of the cytokines that participate in the control of the inflammatory response 14. This cytokine is closely related to interleukin-4 (IL-4) and, for a long time, they were considered to have redundant functions 15. Both cytokines are secreted by T cells, granulocytes, macrophages, and microglia 16,17 and share the same receptors composed of IL-13Rα1 and IL-4Rα subunits 18. Activation of IL-13Rα1/IL-4Rα complex initiates a transduction signal that leads to anti-inflammatory responses 18. Both cytokines use Janus kinases (JAKs) to initiate the signaling and activate the signal transducer and activator of transcription-6 (STAT6) that translocates to the nucleus and regulates gene expression 19. However, IL-4, but not IL-13, also leads to tyrosine phosphorylation of insulin receptor substrate 2 (IRS-2), which in turn activates phosphoinositide 3 kinases (PI3Ks) and protein kinase B (PKB), also known as Akt 18.

In a previous study, we demonstrated that microglia and macrophages adopt a pro-inflammatory phenotype after SCI due, in part, to the insufficient expression of IL-4 in the lesioned spinal cord. We showed that increasing IL-4 levels in the injury site drove microglia and macrophages towards a phenotype conducive for tissue repair, leading to locomotor recovery after SCI 20. However, whether IL-13 exerts similar actions on microglia and macrophages after SCI has not been reported yet. In the present work, we therefore aimed to study the effects of IL-13 on the functional phenotype of microglia and macrophages after SCI.

## Methods

### Approvals

All the experiments were approved by the Ethics Committee on Animal and Human Experimentation from the Universitat Autònoma de Barcelona (CEEAH: 4057) and followed the European Communities Council Directive 2010/63/EU. Methods from each procedure were carried out following approved guidelines and mice were randomized.

### Spinal cord injury

Spinal cord injury procedure was performed according to the described protocol 21. Adult (8-10 weeks old) female C57BL/6J mice from Charles River were anesthetized by intramuscular injection with a mixture of ketamine (90 mg/kg) and xylazine (10 mg/kg). After performing laminectomy at 11^th^ thoracic vertebrae, the exposed spinal cord was contused using the Infinite Horizon Impactor device (Precision Scientific Instrumentation, #IH-0400). A force of 50 kdynes and a tissue displacement of 400-500 µm was applied. Following the directives approved by the Ethics Committee on Animal and Human Experimentation from the Universitat Autònoma de Barcelona, all the SCI experiments were performed in female mice since male rodents show more severe post-operative complications and difficulty with manual bladder expressions, which are required after experimental paralysis. However, there are no sex differences in lesion size or locomotor recovery after SCI in mice or rats 22,23.

### Intraspinal injection of rIL-13 and rIL-4

At 18 or 48 h after SCI, 1 µL containing 100 ng of rIL-13 (BD Bioscience, #554599) or rIL-4 (eBioscience, #574302) was injected into the spinal cord. Phosphate buffer saline (PBS, ThermoFisher Scientific, #10010023) was used as vehicle. Single injections were performed using a 30-µm diameter glass micropipette (Fisher Scientific, #50-821-968) coupled to a 10-µL Hamilton syringe (Sigma-Aldrich, #24530). Glass micropipette was introduced in the middle of the injury epicenter with a depth of 1 mm. Injections were controlled by a 310 Plus automatic injector (KD Scientific, #789311) with a 2 µL/min rate. After injection, the tip of the micropipette was maintained inside the cord tissue for 3 additional minutes to avoid liquid reflux.

### Functional Assessment

Locomotor recovery was evaluated at 1, 3, 5,7, 10, 14, 21, and 28 days post-injury (dpi) on an open-field using the nine-point Basso Mouse Scale (BMS) 24. BMS evaluation was performed by two researchers who were blinded to the experimental groups. A consensus score between two researchers was taken. At least 6 mice per group were used.

### Luminex (bead-based multiplex assay)

Luminex assay was performed according to the described protocol 21. At different time points after injury, mice received an intraperitoneal injection of sodium pentobarbital (Dolethal). Blood was removed by perfusion with 60 mL of 0.9% NaCl in distilled water. 0.6 cm of the spinal cord, centered into the injury site, were taken from each mouse. Samples were frozen in liquid nitrogen and homogenized in extraction buffer using a TissueRuptor (Qiagen, #9002755) and an Ultrasonic Homogenizer (Biologics Inc., #3000). Extraction buffer was prepared following the previous publication 21. Protein was quantified and diluted to 2 mg/µL. Beads and samples were added to the Luminex plate following specific commercial protocols (Invitrogen). After washing, 50 µL of samples were added and incubated overnight at 4ºC. Finally, secondary antibodies were added and the levels of IL-13 and IL-4 were calculated using a MAGPIX Luminex reader (ThermoFisher Scientific). Final values were normalized to the protein concentration of the samples. 4 mice per each time-point were used.

### Flow cytometry

Flow cytometry from the spinal cord was performed according to the described protocol 21. 24 h after intraspinal injection of PBS, rIL-13 or rIL-4, mice received an intraperitoneal injection of sodium pentobarbital (Dolethal). Blood was removed by perfusion with 60 mL of 0.9 % NaCl in distilled water. 0.6 cm of the spinal cord, centered into the injury site, were taken from each mouse. After mechanic and enzymatic disaggregation with collagenase (Sigma-Aldrich, #C-2674) and DNase (Roche, #11284932001), cells in suspension were stained with the following antibodies: CD45-PerCP (BioLegend, #103130), CD11b-PE-Cy7 (BioLegend, #101216), F4/80-APC (eBioscience, #17-4801-82), F4/80-PE (eBioscience, #12-4801-80), F4/80-FITC (MACS Miltenyi, #130-102-988), IL-13Rα1-PE (eBioscience, #12-2130-80), IL-4Rα-APC (MACS Miltenyi, #130-103-348), CD16/32-PE (BioLegend, #101308), CD206-FITC (BioLegend, #141704), Arg1-unconjugated (Santa Cruz, #SC-18354), and iNOS-unconjugated (Abcam, #ab15323). 1:200 dilutions were used for all these antibodies. For unconjugated antibodies, Alexa Fluor 488 (ThermoFisher Scientific, #A1105) or Alexa Fluor 647 (Abcam, #ab150075) secondary antibodies at 1:500 dilutions were used. Proper isotypes for each antibody were selected. Cells were analyzed using the FACSCanto flow cytometer (BD Bioscience). Data were quantified using FlowJo® software. Microglia cells were defined as CD45^low^, CD11b^+^, and F4/80^+^ whereas macrophages were defined as CD45^high^, CD11b^+^, and F4/80^+^ according to previous publications 20,21. Granulocytes, mainly neutrophils, were defined as CD45^high^, CD11b^+^, and F4/80^-^ 20,21. The expression level of each marker was calculated by dividing the number of positive cells from each marker over the total of CD45^+^ cells. 4 mice per group were used.

### Histology

At the end of the functional evaluation, mice were perfused with 4% paraformaldehyde (Sigma-Aldrich, #F8775) in 0.1M phosphate buffer. 0.6 cm of the spinal cord, centered into the lesion site, were removed from each mouse and cryoprotected with 30% sucrose in 0.1 M of PBS at 4 ºC. Samples with 10-µm thick were cut in the cryostat and picked up with a glass slide. Samples were arranged following a serial distribution. Adjacent sections on the same slide were 100 µm apart. After graded dehydration, sections were placed in a 1 mg/mL Luxol Fast Blue (LFB, Sigma-Aldrich, #S3382) solution in 95% ethanol and 0.05% acetic acid and left overnight at 37 ºC. Sections were then washed in 95% ethanol and cleared with 0.5 mg/mL of Li_2_CO_3_ (Sigma-Aldrich, #255823) in distilled water for 1.5 min. After several washes, sections were dehydrated and mounted in DPX mounting media (Sigma-Aldrich, #44581). After fixation, the epicenter of the contusion was localized by determining the tissue section with the lowest LFB stained area. NIH ImageJ software was used to perform the quantifications. 6-8 mice per group were used.

### Cell sorting and RNA extraction

The flow cytometry protocol, with slight modifications, was used for cell sorting. Myelin was removed from the spinal cord cell suspension using myelin removal beads (MACS Miltenyi, #130-096-731) and LS MACS columns (MACS Miltenyi, #130-096-731) following the manufacture's recommendations. Afterward, cells were stained with CD45-PerCP, CD11b-PE-Cy7, and F4/80-PE antibodies and sorted using the FACSJazz (BD Bioscience). 1 drop pure method was used. A drop frequency of 39.05 kHz and a sheath pressure of 27 psi were established. Sort rate varied between 100-150 cells/sec depending on the sample since treatments can alter cell density. Populations were gated as previously described using the basis of FSC and SSC, CD45-PerCP, CD11b-PE-Cy7, and F4/80-PE antibodies. From 9,000 to 73,000 events of macrophages or microglia were obtained from each mouse. The purity after sorting was 99%. Cells from sorting were collected in Eppendorf tubes with 350 µL of ice-cold RLT buffer (Qiagen, #74004). RNA extraction was performed using the RNeasy Micro Kit (Qiagen, #74004) following the manufacturer's recommendations. RNA was stored at -80 ºC until processing. 3 mice per group were used.

### Low-input RNA-seq

RNA sequencing libraries were prepared following the SMARTseq2 protocol 25 with some modifications. Briefly, RNA was quantified using the Qubit RNA HS Assay Kit (ThermoFisher Scientific, #Q32852) and the input material used for the initial cDNA synthesis varied in function of the available sample concentration (0.12ng-10ng). Reverse transcription was performed using SuperScrpit II (Invitrogen, #18064022) in the presence of oligo-dT30VN, template-switching oligonucleotides (1 µM) and betaine (1M). The cDNA was amplified using the KAPA Hifi Hotstart ReadyMix (Kappa Biosystems, #7958927001), 100 nM ISPCR primer, and 15 cycles of amplification. The amplified cDNA (200 ng) was fragmented using Nextera® XT (Illumina, #FC-131) and amplified for 10-12 cycles with indexed Nextera® PCR primers. The Nextera® library was purified twice with Agencourt Ampure XP beads (0.8:1 ratio). The libraries were sequenced on HiSeq2500 (Illumina, Inc) in paired-end mode with a read length of 2x76bp using TruSeq SBS Kit v4 and Nextera XT Index Kit of 8bp+8bp. Image analysis, base calling and quality scoring of the run were processed using the manufacturer's software Real Time Analysis (RTA 1.18.66.3) and followed by generation of FASTQ sequence files by CASAVA. The data generated in this publication have been deposited in NCBI's Gene Expression Omnibus 26 and are accessible through GEO Series accession number GSE158510 (https://www.ncbi.nlm.nih.gov/geo/query/acc.cgi?acc=GSE158510).

### RNA-seq processing and data analysis

RNA-seq paired-end reads were mapped against the mouse reference genome (GRCm38) using STAR version 2.5.3a 27 with ENCODE parameters for long RNA. Annotated genes were quantified using RSEM version 1.3.0 with default parameters 28. Differential expression analysis was performed with DESeq2 version 1.18.1 29 for the microglia and macrophages experiments separately. Principal component analysis was done using the top 500 most variable genes with the 'prcomp' R function and 'ggplot2' R library. Heatmap with the top 50 differentially expressed genes for each comparison was performed with the 'pheatmap' R package with the 'rlog' transformed counts from DESeq2 and scaling by row. KEGG pathway representation was performed with the DAVID db version 6 30,31. Normalized expression values for specific markers were also used to evaluate the levels of damage-associated genes.

### Cell metabolism

24 h after intraspinal injections of PBS, rIL-13, or rIL-4, mice were deeply anesthetized with sodium pentobarbital and spinal cord cell suspensions were generated as previously described 21. Cells were incubated with CD11b magnetic microbeads (MACS Miltenyi, #130-049-601) and then loaded into LS MACS columns (MACS Miltenyi, #130-042-401) following the manufacturer's guidelines. Myeloid cells were purified and quantified in the Neubauer Chamber. 200,000 cells per mice and well were plated in a XFp cell culture miniplate (Agilent, #103025-100) following the specific manufacturer's protocol for cell suspensions. Seahorse XF Base Medium (Agilent, #103335-100) supplemented with 1 mM pyruvate (Sigma-Aldrich, #S8636), 2 mM glutamine (Sigma-Aldrich, #G-7513), and 25 mM glucose (Sigma-Aldrich, #G-7021) was used as a measurement medium. A Mixture of 1 µM oligomycin and 1.5 µM carbonyl cyanide-4-(trifluoromethoxy) phenylhydrazone (FCCP) (Agilent, #103275-100) were loaded into the proper cartridge (Agilent, #103022-100). Measurements of extracellular acidification rate (ECR), a hallmark of glycolysis based on the rate of increase in proton concentration (or decrease in pH), and oxygen consumption rate (OCR), a hallmark of mitochondrial respiration based on the rate of oxygen concentration in the assay medium, from live cells were performed using the Seahorse XFp Cell Energy Phenotype protocol in a XFp Analyzer (Agilent, #103275-100). 3 measures were taken under basal conditions and 5 measures after exposition to oligomycin and FCCP stressors. 3 mice per group were used.

### Cell culture

Bone marrow-derived macrophages (BMDM) were cultured according to described protocols 32. Briefly, mice were euthanatized and cells from femurs were collected. 4 x 10^5^ cells were plated per sterile plastic petri dish in 10 mL of DMEM/F-12 (ThermoFisher Scientific, #11320033) supplemented with 10% fetal bovine serum (Sigma-Aldrich, #F2442), 1% penicillin/streptomycin (Sigma-Aldrich, #P4333), and 100 ng/mL of recombinant M-CSF (R&D Systems, #416-ML). Cells were incubated at 37 ºC and 5% CO_2_ and the medium was changed at days 3, 5 and 7. At day 7, cells were exposed to conditioning conditions: (i) 100 ng/mL of LPS (Sigma-Aldrich, #L2880) + 20 ng/mL of rINFγ (R&D Systems, #485-MI), (ii) 20 ng/mL of rIL-4 (eBioscience), (iii) 20 ng/mL of rIL-13, (iv) 7.9 µg/mL of Oligomycin (Sigma-Aldrich, #75351) + 2.1 µg/mL of Antimycin (Sigma-Aldrich, #A8674), (v) 20 ng/mL of rIL-4 + 7.9 µg/mL of Oligomycin + 2.1 µg/mL of Antimycin, and (vi) 20 ng/mL of rIL-13 + 7.9 µg/mL of Oligomycin + 2.1 µg/mL of Antimycin. Concentrations were based on previous publications 33. After 24 h, supernatants (conditioned mediums) were collected and stored at -20 ºC until use.

Motor neuron-like NSC-34 cells were cultured in DMEM (ThermoFisher Scientific, #10566016) supplemented with 10% fetal bovine serum and 1% penicillin/streptomycin (Sigma-Aldrich, #P-0781) in a 37 ºC and 5% CO_2_ incubator.

### MTT viability assay

To perform the viability assay, NSC-34 cells were harvested and plated at 10^5^ cells/mL in a 96-well plate. 24 h later, cell medium was completely removed and substituted with the different macrophage-conditioned mediums. After 24 h, cell viability was analyzed by incubation with 5 mg/mL of 3-(4,5-dimethylthiazol-2-yl)-2,5-diphenyltetrazolium bromide (MTT, ThermoFisher Scientific, #M6494) solution for 1 h at 37 °C. Afterwards, MTT salts were dissolved in DMSO (Sigma-Aldrich, #472301) and optical density (OD) at 562 nm was measured using an Elx800 microplate reader (BioTek). The percentage of surviving cells was determined by dividing the optical density of each condition with the control medium. 5 biological replicates per condition were used.

### Immunofluorescence

NSC-34 cells stimulated with the different macrophage-conditioned medium for 24 h were washed with ice-cold PBS and fixed with 4% paraformaldehyde (Sigma-Aldrich) in 0.1M phosphate buffer. Fixed cells were treated with TBS with 0.3% Triton X-100 (Sigma-Aldrich, #9002-93-1) and 10% normal donkey serum (Merck Millipore, #566460) for 1 h. Cells were then incubated with 1:500 mouse anti-Tubulin β3 monoclonal antibody (Biolegend, #801213) o/n at 4 °C. Cells were then stained with 1:200 Alexa Fluor 488 anti-mouse antibody (Invitrogen, #A28175) for 1 h at room temperature and finally mounted in Mowiol mounting media (Sigma-Aldrich, #81381). Photographs were taken using an Olympus DP76 digital camera attached to the microscope (Olympus BX51).

### Statistics

All the analyses were conducted by GraphPad Prism 8 software. The Kolmogorov-Smirnov test was used to test normality. BMS score and cell metabolic assays were analyzed using two-way repeated measures ANOVA with Bonferroni's post hoc correction. Cytokine levels from Luminex and absorbance form MTT assay were analyzed using one-way ANOVA with Dunnett's post hoc correction. Flow cytometry data were analyzed using one-way ANOVA with Tukey's post hoc correction. Normalized expression values for damage-associated genes were also evaluated using the same method. Myelin sparing was analyzed using multiple t-test comparisons with Holm-Sidak's post hoc correction. Results were expressed as mean ± SEM and differences were considered significant at p<0.05.

## Results

### SCI induces changes in the levels of IL-13 and its receptors

**Figure 1:**
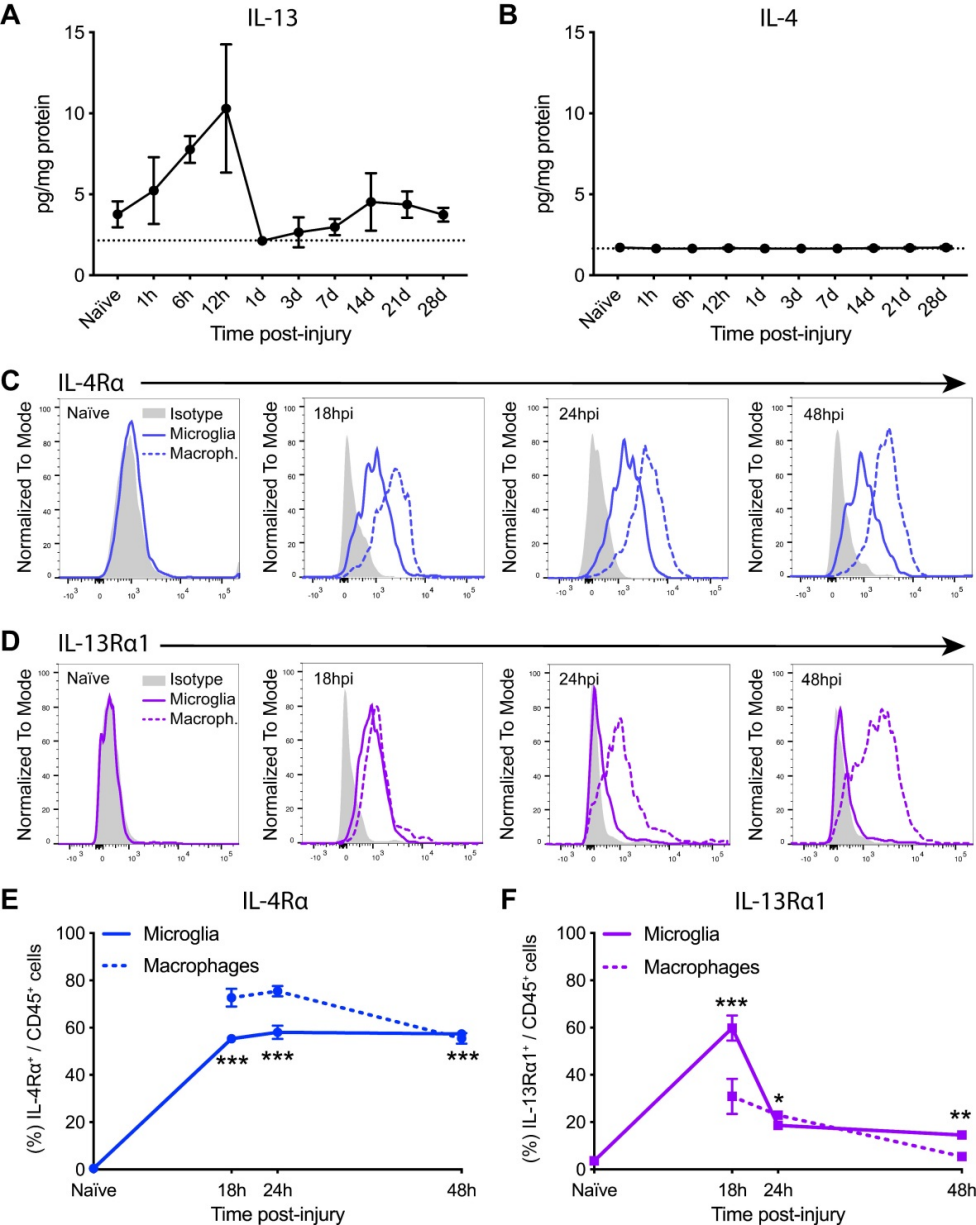
** Injury to the spinal cord induces changes in the levels of IL-13 and IL-4Rα/IL-13Rα1 receptor subunit. (A and B)** Levels of IL-13 (A) and IL-4 (B) in the spinal cord parenchyma were measured for 28 days after injury. Dashed line marks the detection limit of the Luminex assay (2.15 pg/mg protein for IL-13 and 1.44 pg/mg protein for IL-4). **(C and D)** Representative flow cytometry histograms of IL-4Rα (C) and IL-13Rα1 (D) in microglia (solid line) and macrophages (dashed line) after SCI. **(E and F)** Percentage of microglia and macrophages expressing IL-4Rα (E) and IL-13Rα1 (F). Data are represented as Mean ± SEM. N = 4 per time point. ** p < 0.01, *** p < 0.001 vs naïve. One-way ANOVA with Dunnett's post hoc correction for (A), (B), (E), and (F).

Since IL-13 has effects on immune cells that are closely related to those exerted by IL-4, we assessed the dynamic changes of IL-13 protein levels in the spinal cord parenchyma after contusion injury. Luminex assay revealed that IL-13 was expressed at very low levels in the spinal cord under normal physiological conditions (Figure 1A). After SCI, protein levels of IL-13 showed a tendency, although not significant, to increase but only for the first 12 h. After 24 h, IL-13 returned to basal levels (Figure 1A). In agreement with our previous publication 20, IL-4 protein levels were not up-regulated for the first 28 days after SCI (Figure 1B).

We then analyzed the expression of IL-13Rα1 and IL-4Rα receptor subunits in microglia and macrophages for the first 48 h after SCI (Figure S1). Flow cytometry analysis revealed that microglia in the spinal cord did not express any of these subunits at physiological conditions (Figure 1C-F). At 18 h post-injury, IL-4Rα was found in ~55% of microglia and ~70% of macrophages (Figure 1C,E) whereas IL-13Rα1 was found in ~60% of microglia and ~30% of macrophages (Figure 1D,F). From 24 to 48 h after injury, the presence of IL-4Rα was found in ~50% of both myeloid cell subsets (Figure 1C,E) whereas the expression of IL-13Rα1 was observed in ~25% of microglia and macrophages (Figure 1D,F).

### rIL-13 increases infiltration and anti-inflammatory polarity of macrophages

Since levels of IL-13 were very low (Figure 1A) but receptors were available (Figure 1C-D) after SCI, we hypothesized that increasing the bioavailability of IL-13 at the lesion site may skew microglia and macrophages towards an anti-inflammatory phenotype. Based on our previous publication with rIL-4 20, we administered rIL-13 at the injury site at 48 h after SCI and evaluated its effect on immune cell counts and polarization after 24 h. We also injected rIL-4 in a group as positive control. PBS was used as negative control.

**Figure 2:**
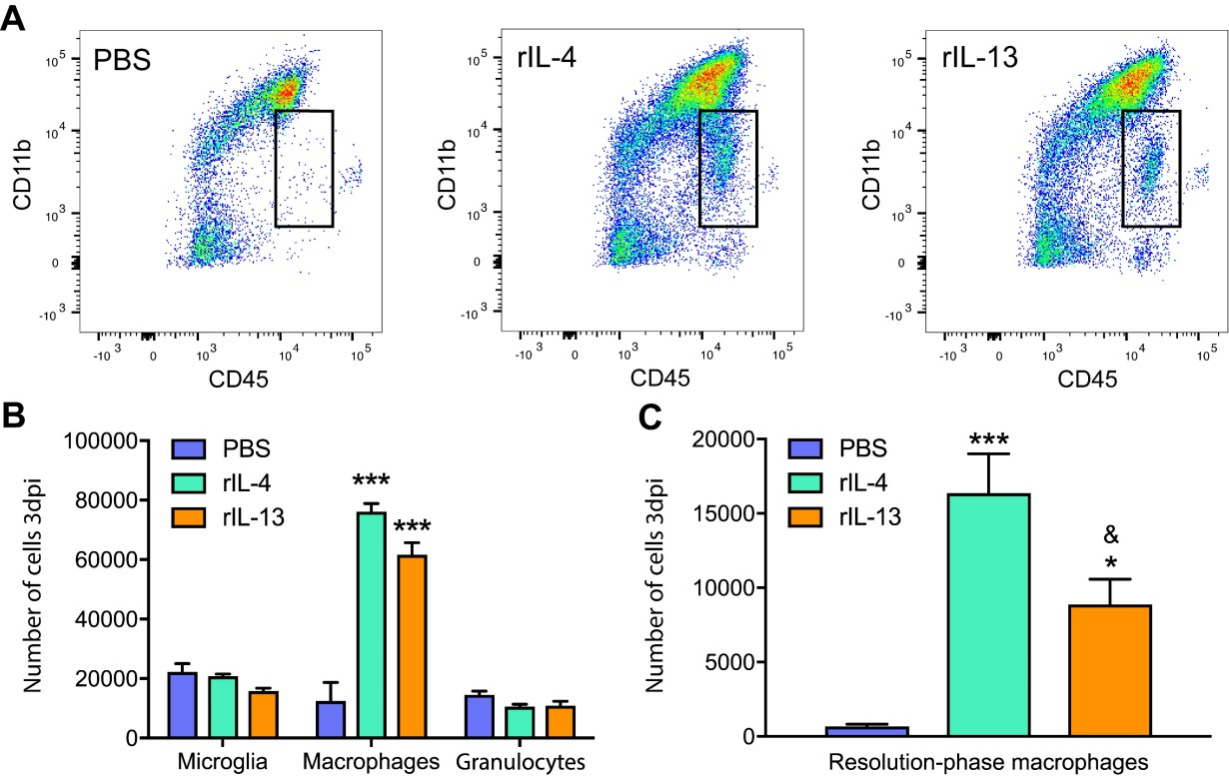
** Administration of IL-13 or rIL-4 increases the infiltration of macrophages at 3 days after SCI. (A)** Representative flow cytometry density plots showing CD45^+^ and CD11b^+^ cells in the spinal cord after treatment with PBS, rIL-4, and rIL-13. Note that rIL-4 and rIL-13 led to the appearance of resolution-phase macrophages (square) absent in PBS. **(B)** Plot showing the counts of microglia, macrophages, and granulocytes. **(C)** Plot showing the counts of resolution phase-macrophages. Data are presented as mean ± SEM. N = 4 per group. * p < 0.05, *** p < 0.001 against PBS. & p < 0.05 against rIL-4. One-way ANOVA with Tukey's post hoc test.

We found that rIL-13 led to similar effects than rIL-4 on macrophages and microglia counts (Figure 2A). Both cytokines significantly increased the number of infiltrated macrophages into the injured spinal cord but did not alter the number of microglia or granulocytes (Figure 2B). rIL-13 and rIL-4 also induced the appearance of a population of myeloid cells (CD45^high^, CD11^low^) that was minimally detectable in the lesioned spinal cord of mice treated with PBS (Figure 2A). We previously shown that rIL-4 induced the appearance of this myeloid cell subset in the injured spinal cord and that they were phenotypically compatible with resolution phase macrophages 20. Nevertheless, the counts of this resolution phase macrophages-like cells in the spinal cord was ~2 fold increased with rIL-4 relative to rIL-13 (Figure 2C).

**Figure 3:**
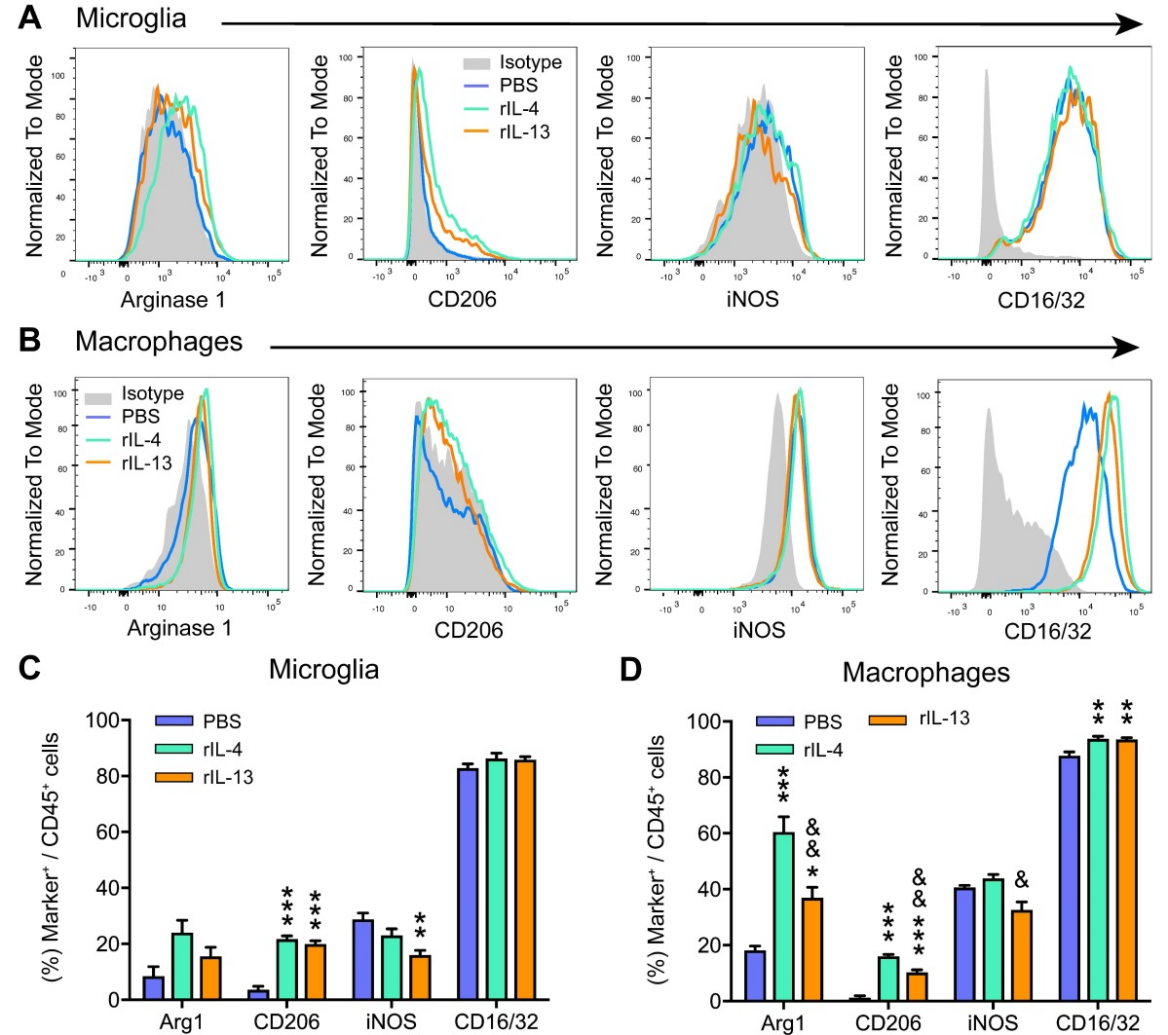
** rIL-13 and rIL-4 induce macrophages and microglia towards an anti-inflammatory phenotype. (A and B)** Representative flow cytometry histograms of the key markers used to define the polarization state of microglia (A) and macrophages (B) at 3dpi. **(C and D)** Graphs showing the percentage of microglia (C) and macrophages (D) expressing the pro- and anti-inflammatory markers at 3 dpi. Data are represented as Mean ± SEM. N = 4 per group. * p < 0.05, ** p < 0.01, and *** p < 0.001 against PBS. & p < 0.05 and && p < 0.01 against rIL-4. One-way ANOVA with Tukey's post hoc correction.

We then investigated whether rIL-13 altered the polarization of macrophages and microglia in the injured spinal cord by evaluating the levels of pro-inflammatory (iNOS and CD16/32) and anti-inflammatory (Arg1 and CD206) markers. In agreement with previous works 20,34, we found only a small proportion (<20%) of microglia (Figure 3A,C) and macrophages (Figure 3B,D) expressing the anti-inflammatory markers, CD206 and Arg1, at 3 days post-lesion in PBS-mice. In contrast, the pro-inflammatory markers, iNOS and CD16/32, were highly expressed in both myeloid cell subsets. Treatment with rIL-13 and rIL-4 significantly increased the proportion of microglia and macrophages expressing Arg1 and CD206, indicating that both cytokines induced a change towards anti-inflammatory phenotype. Comparatively, rIL-4 led to slightly, but significant, greater expression of anti-inflammatory markers than rIL-13 in microglia (Figure 3C) but not in macrophages (Figure 3D). Contrarywise, rIL-13 reduced the expression of iNOS in microglia and tended to decrease its levels in macrophages, while rIL-4 failed to alter the expression of this pro-inflammatory marker in both myeloid cell subsets (Figure 3C,D). Indeed, the expression of iNOS in macrophages was slightly, but significant, reduced by rIL-13 as compared to rIL-4 (Figure 3C,D).

### rIL-13 fails to promote functional and histological outcomes after SCI

**Figure 4:**
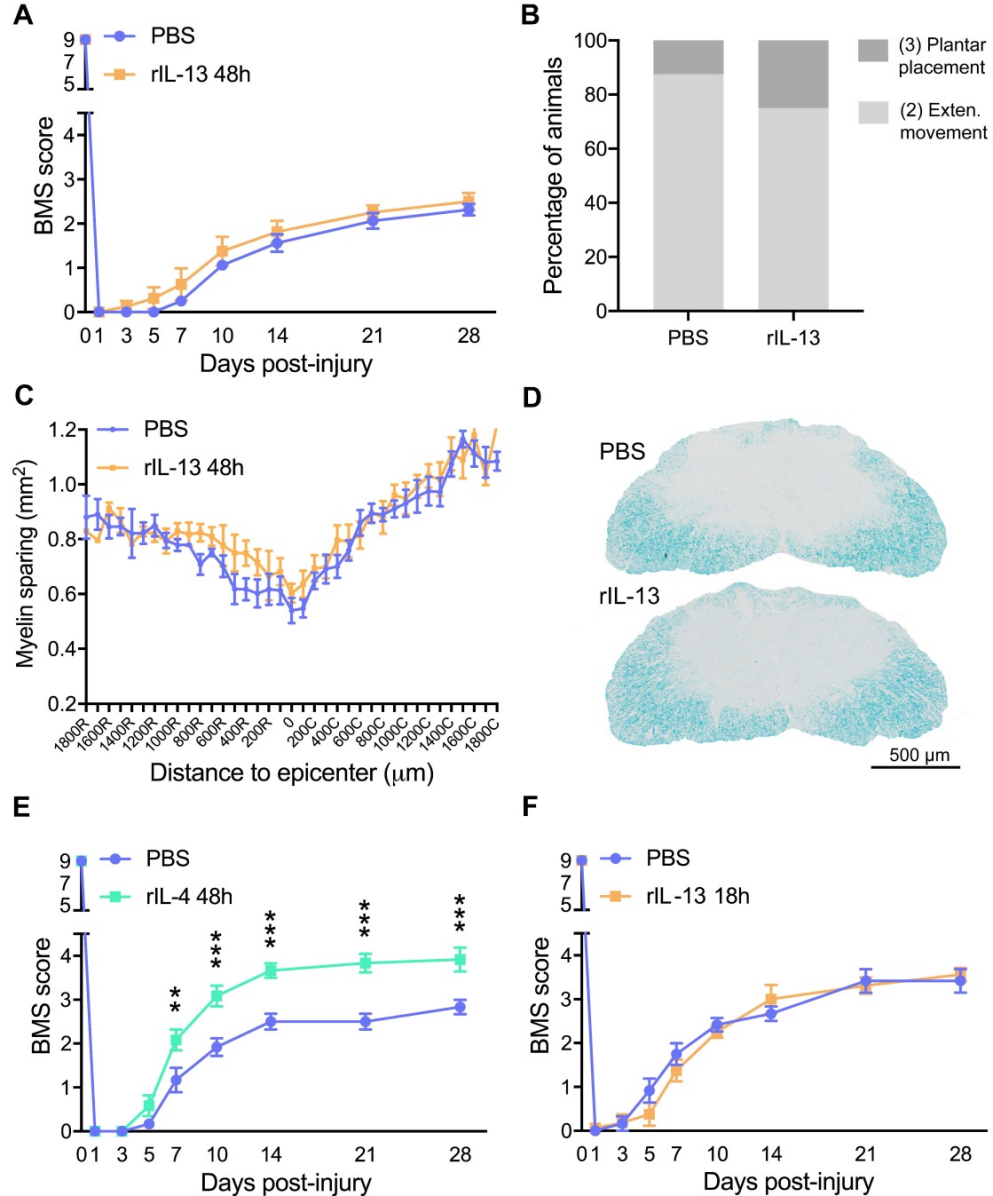
** Administration of rIL-13 does not improve functional or histological outcomes after SCI. (A)** Plot showing the BMS score of mice over the first 28 days post-injury. **(B)** Graph showing the functional distribution of mice at 28 dpi. BMS values of each type of movement are indicated in parenthesis. **(C)** Graph showing the quantification of myelin preservation in tissue sections stained with LFB. (D) Representative micrographs showing myelin sparing at the injury epicenter in rIL-13 and PBS-injected mice. **(E, F)** Graph showing locomotor recovery of mice injected with rIL-4 at 48 h after injury (E) or rIL-13 at 18 h after lesion (F). Data are represented as mean ± SEM. N = 8 per group for (A, B, and C); N = 6 per group for (E); N = 6 PBS and N = 8 rIL-13 for (F). ** p < 0.01 and *** p < 0.001. Two-way RM-ANOVA with Bonferroni's post hoc correction for (A, E, and F) and multiple t-test comparisons with Holm-Sidak's post hoc correction for (C).

Since rIL-13 induced an anti-inflammatory/pro-repair phenotype of macrophages and microglia when injected at 48 h following injury, we then studied whether these changes were associated with greater functional and histopathological outcomes. Unexpectedly, we found that rIL-13 did not lead to any locomotor improvement for the first 28 days after injury (Figure 4A). At the end of the follow up, ~80% of the mice from PBS and rIL-13 groups showed extensive ankle movement and only a few of them reached plantar placement (final BMS score ~2.4 for both groups) (Figure 4B). Similarly, histological analysis of the spinal cord from mice injected with rIL-13 did not reveal any enhancement in the myelinated area either at the epicenter of the lesion or in adjacent areas (Figure 4C,D). Contrarywise, and in line with our previous report 20, administration of rIL-4 used as positive control resulted in significant improvement of functional recovery (Figure 4E).

Then, we evaluated the effects of intraspinal injection rIL-13 when given at 18 h post-injury, which coincides with the peak of IL-13Rα1 expression in macrophages and microglia (Figure 1F). As happened at 48 h, administration of rIL-13 at 18h post-injury did not lead to locomotor recovery after SCI (Figure 4F). These results revealed that, although treatment of rIL-13 and rIL-4 exerted similar effects on microglia and macrophage counts and phenotype, only rIL-4 was able to promote beneficial functional effects after SCI.

### rIL-13 and rIL-4 induce different transcriptomic profiles in microglia

We then sought to investigate the different mechanisms modulated by rIL-4 and rIL-13 in macrophages and microglia that could be related to functional recovery after SCI. For this purpose, we intraspinally injected mice with PBS, rIL-13 or rIL-4 at 48 h after contusion injury. 24 h later, we purified microglia and macrophages from the lesioned spinal cord and performed an RNA sequencing (RNA-seq) analysis.

**Figure 5:**
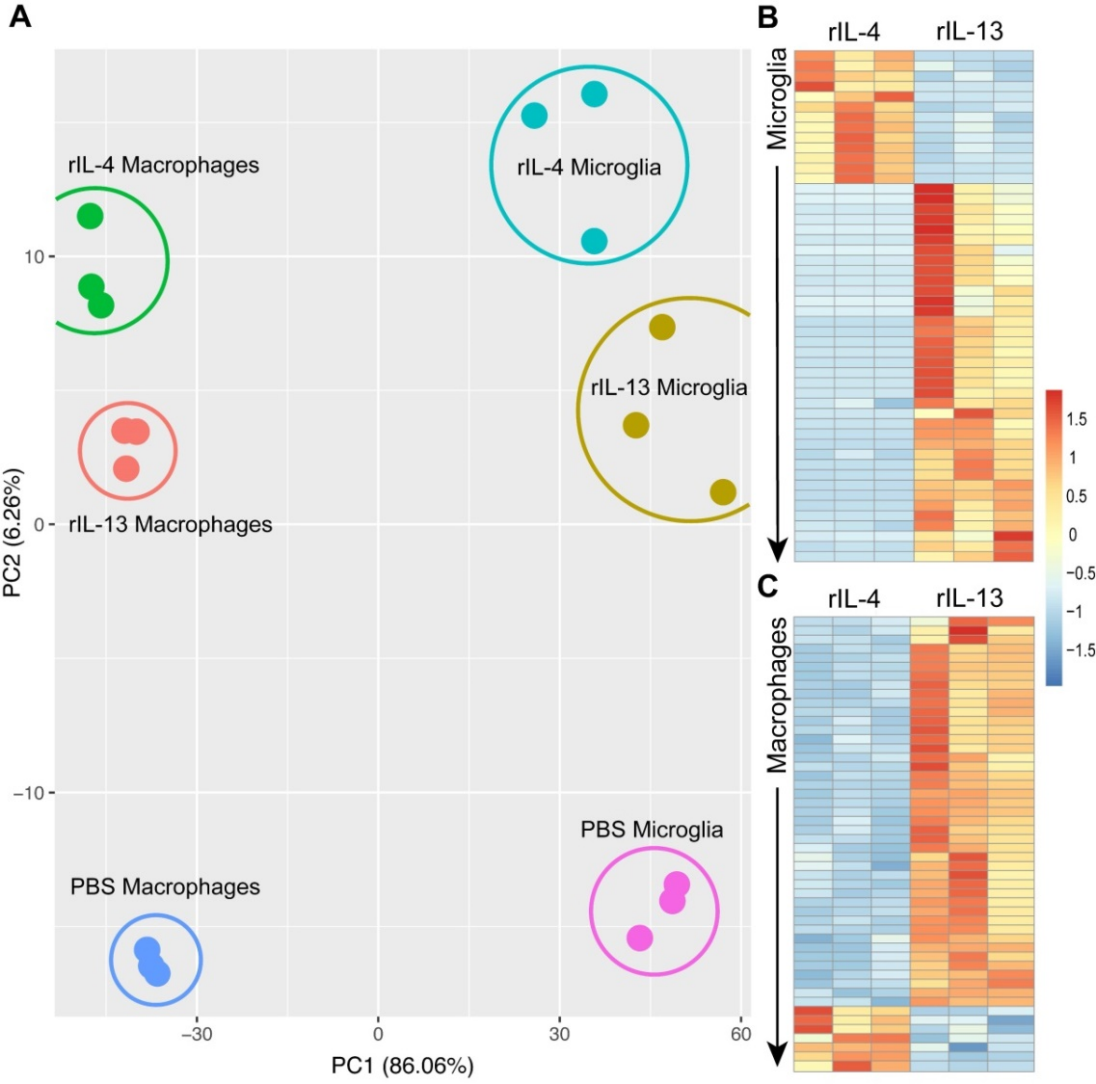
**Transcriptomic profile of macrophages and microglia change after treatment with PBS, rIL-13 or IL-4. (A)** PCA of total of 18 samples analyzed. PCA1 and PCA2 explained the 86.06% and 6.26%, respectively, of the total variance. Microglia and macrophages sorted from the injured spinal cord injected with PBS were used as a reference for the untreated control conditions. **(B and C)** Heatmap of top 50 differentially expressed genes (adjusted p-value < 0.05) between rIL-13 and rIL-4 in macrophages (B) and microglia (C). Upregulated genes are marked in warm colors whereas downregulated genes are marked in cold colors. Detailed version of (B) and (C) can be observed at Figure S2 and Figure S5, respectively.

Principal Component Analysis (PCA) revealed that rIL-4 and rIL-13 mediated different gene signatures on these immune cells (Figure 5A). Although the effects of these cytokines on the polarization state of microglia and macrophages was very similar, they induce different responses at gene level. To identify specific changes caused by rIL-4 and rIL-13 in microglia and macrophages, we focused on genes that were differentially expressed (DE) (adjusted p-value < 0.05) in these two myeloid cell populations by the effects of these cytokines.

We identified 477 genes that were DE in microglial cells after rIL-4 and rIL-13 injection. Among them, 352 were up-regulated by rIL-13 whereas 125 were up-regulated by rIL-4. Similarly, in the list of the top 50 DE genes in microglial cells, only as small proportion (13 out 50) were up-regulated in rIL-4 relative to rIL-13 (Figure 5B, S2). Among them, 6 were related to elements of the mitochondria respiratory chain, suggesting that rIL-4 could foster mitochondrial respiration in microglia in comparison to rIL-13. We also performed gene ontology (GO) analysis (Figure S3) to evaluate multiple biological processes (Figure S3A), molecular functions (Figure S3B), and cellular components (Figure S3C) that could be differentially altered between rIL-13 and rIL-4 in microglia. Again, several events related to cell metabolism were found to be differently induced. In this line, oxidative phosphorylation appeared as one of the three Kyoto Encyclopedia of Genes and Genomes (KEGG) defined pathways differentially enriched in microglia by rIL-4 in comparison to rIL-13 (Figure S3D, S4).

**Figure 6:**
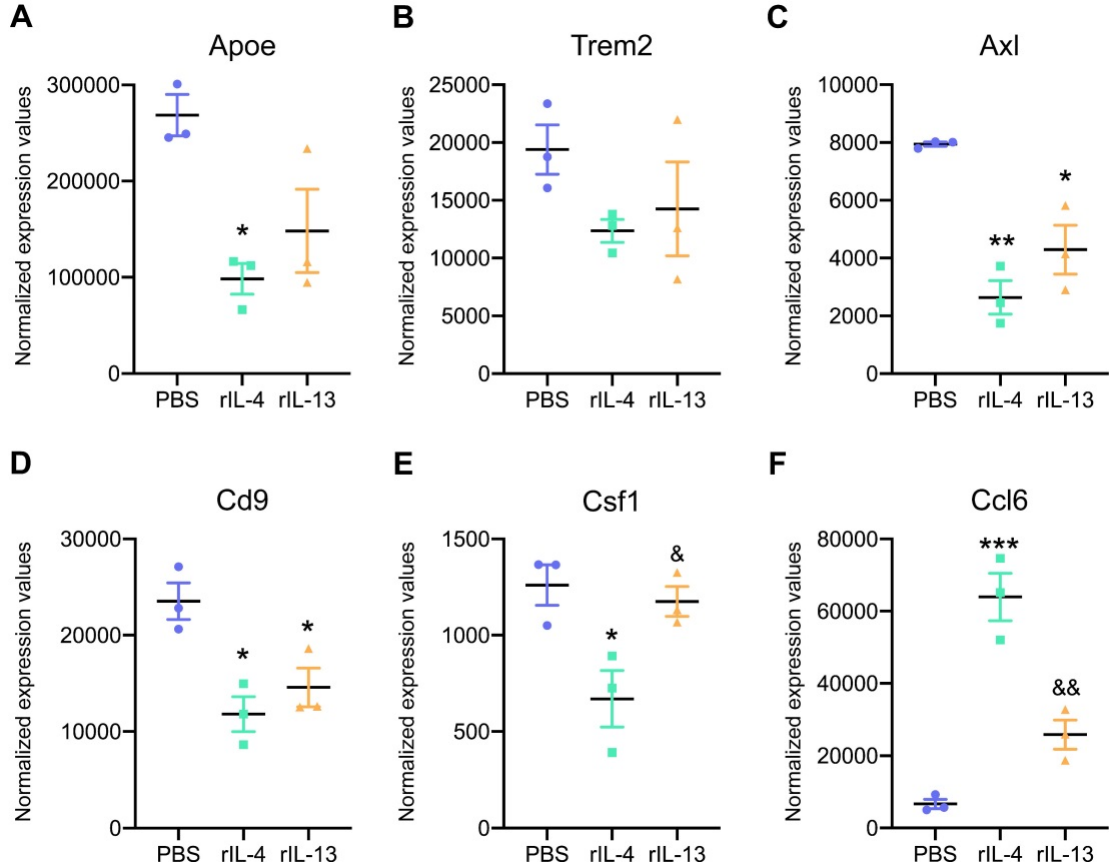
** Expression levels of key damage-associated genes in microglia (DAM) was reduced by rIL-4 and rIL-13.** Normalized expression values for *Apoe* (**A**), *Trem2* (**B**), *Axl* (**C**), *Cd9* (**D**), *Ccl6* (**E**), and *Csf1* (**F**) in microglia from injured spinal cord after treatment with PBS (blue), rIL-4 (green), and rIL-13 (orange). N = 3 per group. * p < 0.05, ** p < 0.01, and *** p < 0.001 against PBS. & p < 0.05 and && p < 0.01 against rIL-4. One-way ANOVA with Tukey's post hoc correction.

We then studied the expression of key genes that are upregulated in damage-associated microglia (DAM) and that have been recently described in Alzheimer's disease and other neurological conditions 35,36. We found enrichment for DAM markers, such as, *ApoE*, *Trem2*, *Axl*, *Cd9*, *Csf1*, and *Ccl6* in microglial cells after SCI (Figure 6). Intraspinal injection of rIL-13 and rIL-4 reduced the expression of most of these genes, except for *Ccl6*, that was increased by both cytokines (Figure 6F). For the rest of the genes related to DAM, rIL-4 seemed to induce a stronger reduction than rIL-13 (Figure 6A-E).

### rIL-13 and rIL-4 induce different transcriptomic profiles in macrophages

We also assessed the effects of rIL-4 and rIL-13 in macrophages after SCI (Figure 6C, S5). We identified 415 that were DE in macrophages after rIL-13 and rIL-4 injection. Like in microglia, rIL-13 up-regulated most of these genes (251) relative to rIL-4 in macrophages. Again, among the genes that were significantly up-regulated by rIL-4, many of them were related to mitochondrial respiration such as *Mt-co1*, *Mt-nd4*, *Mt-cytb*. Gene ontologies (GO) (Figure S6) revealed multiple biological processes (Figure S6A), molecular functions (Figure S6B), and cellular components (Figure S6C) that were differentially altered in macrophages by rIL-13 and rIL-4. They included events related to cell adhesion, cell proliferation, but also cell metabolism. These RNA-seq data suggest that rIL-4 might favor a more oxidative metabolism in microglia and macrophages as compared to rIL-13.

### IL-13 and rIL-4 induce different metabolic states in immune cells

Cell metabolism plays an important role in regulating immune cell activity 37. Since our RNA-seq analysis suggested that rIL-4 and rIL-13 could promote differences on the immune metabolism (Figure S3,S6), we assessed the energetic phenotype induced by these cytokines in these two immune cell subsets by Seahorse technology.

With this aim, we intraspinally injected PBS, rIL-13 or rIL-4 at 48 h post-injury. 24 h later, we magnetically isolated CD11b^+^ cells (myeloid cells). Magnetic isolation of myeloid cells from the spinal cord instead of cell sorting of microglia and macrophages was done since the latter procedure is longer and the time required may vary considerably between samples. This is important since we found that the metabolic state of the microglia and macrophages changes with the time taken for isolation of the cells and introduces large variability between samples even from the same experimental group (data not shown). Immediately after isolation of myeloid cells, we assessed their extracellular acidification rate (ECAR; an index of glycolysis) and oxygen consumption rate (OCR; an index of oxidative phosphorylation) under basal conditions and upon simultaneous addition of the mitochondria perturbing agents oligomycin and FCCP.

**Figure 7:**
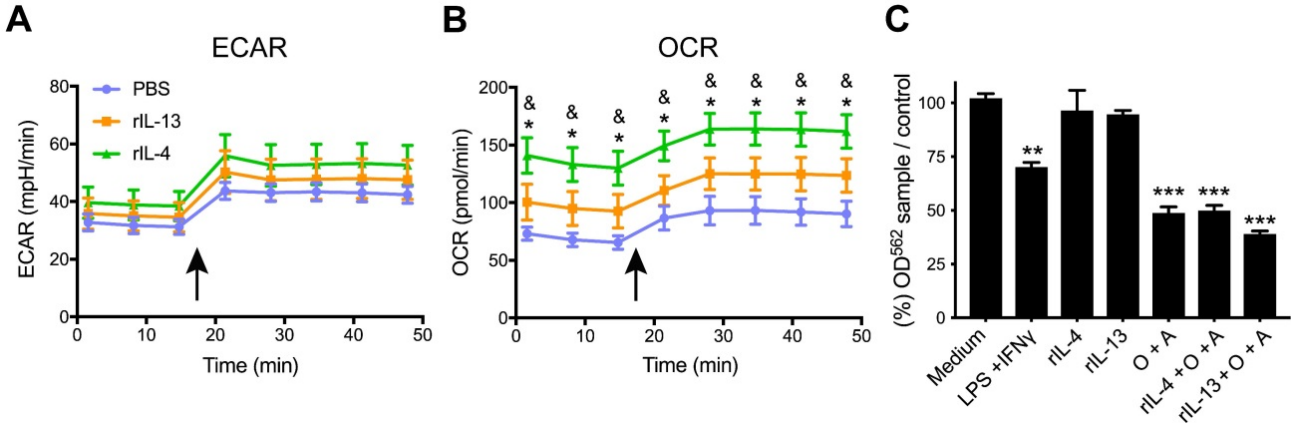
** rIL-4 induces a shift in the energetic phenotype of myeloid cells isolated from the spinal cord, from glycolysis to oxidative phosphorylation. (A and B)** Plot showing the changes in ECAR (A) and OCR (B) values in the assay medium over 50 min. Oligomycin and FCCP was added after 15 min (arrow) to induce stressed phenotype and determine glycolytic and oxidative potentials, respectively. **(C)** Effects of BMDMs on NSC-34 cells viability. MTT assay showing the cell viability of NSC-34 cells after exposition to conditioning medium from BMDMs. Oligomycin and antimycin (O+A) were used to block the oxidative phosphorylation. Data are represented as mean ± SEM. N = 3 per group for (A) and (B) and N = 5 per group for (C). * p < 0.05 against PBS. & p < 0.05 against rIL-13 based on two-way RM ANOVA with Bonferroni's post hoc correction for (A) and (B). ** p < 0.01, and *** p < 0.001 against control medium based on one-way ANOVA with Dunnett's post hoc correction for (C).

We found that myeloid cells isolated from PBS-injected contused spinal cords showed basal OCR/ECAR ratio of ~2, as indicator of cellular metabolism (Figure 7A,B). Upon simultaneous exposition to oligomycin and FCCP stressors, myeloid cells increased both glycolysis and mitochondrial respiration, as indicated by the ECAR and OCR values, respectively. However, the OCR/ECAR ratio remained unchanged. Myeloid cells isolated from the injured spinal cord injected with rIL-13 showed basal ECAR values comparable to those observed in myeloid cells from the lesioned spinal cord injected with PBS. Basal OCR values were increased but not significantly (Figure 7A,B). Oligomycin and FCCP also increased glycolysis and oxidative phosphorylation in myeloid cells from rIL-13 treated mice, but no significant differences were detected with those isolated from the PBS group (Figure 7A,B). Similar to rIL-13, rIL-4 did not alter the glycolytic rate in the myeloid cells from the injured spinal cord as indicated by the basal ECAR values (Figure 7A). However, rIL-4 markedly increased basal OCR values as compared to PBS and rIL-13 groups (Figure 7B) suggesting that this cytokine boosted the oxidative phosphorylation in myeloid cells in the injured spinal cord. rIL-4 increased the OCR/ECAR ratio by ~100% and 50% as compared to myeloid cells from PBS and rIL-13, respectively. These data confirm the results predicted by the RNA-seq analysis and demonstrate that rIL-4 has a greater impact in shifting energy production in myeloid cells from glycolytic towards oxidative phosphorylation after damage.

### Metabolic changes in myeloid cells affect neuronal survival

Having demonstrated that rIL-4 favors oxidative metabolism in myeloid cells after SCI, we next sought to assess the effects of this metabolic switch on neuronal survival. For this purpose, we exposed bone marrow-derived macrophages (BMDM) to: (1) IFNγ+LPS to induce M1 state, (2) rIL-4 or rIL-13 to induce M2 state, (3) oligomycin+antimycin (O+A) to inhibit mitochondrial respiration and foster glycolytic metabolism, and (4) rIL-4 or rIL-13 in combination with O+A to inhibit mitochondrial respiration in M2 macrophages. After 24 h of stimulation, conditions media was collected and placed in the presence of a motoneuron-like cell line (NSC-34) to assess the effects on viability.

As expected, stimulation of BMDM with rIL-4 or rIL-13 enhanced M2 polarization based on the levels of CD206 (Figure S7A). Exposure of NSC-34 cells to the conditioned medium derived from BMDM stimulated with LPS and INFγ reduced cell viability in ~30% according the MTT assay (Figure 6C,S7B). The conditioned medium of BMDM stimulated with rIL-4 or rIL-13 did not have any impact on NSC-34 survival, indicating that M1, but not M2, macrophages are neurotoxic (Figure 7C,S7B). Inhibition of oxidative phosphorylation by oligomycin and antimycin also reduced the survival of NSC-34 cells, to a similar extent to that observed after LPS+INFγ exposition. This suggests that BMDM become neurotoxic when glycolysis is promoted (Figure 7C,S7B). Finally, we observed that M2 polarized BMDM were also cytotoxic when mitochondrial respiration was inhibited (Figure 7C,S7B). These findings indicate that macrophages do not mediate cytotoxic responses when rely on oxidative metabolisms and support that the beneficial effects of rIL-4 after SCI are due, at least in part, to metabolic reprogramming of myeloid cells towards oxidative metabolism against glycolysis. Although macrophages stimulated with IL-13 are more dependent on oxidative phosphorylation in cell culture conditions 38, IL-13 did not foster this metabolic shift in SCI, which may explain, in part, the failure of this cytokine to promote beneficial actions in SCI.

## Discussion

Spinal cord injury elicits an inflammatory response produced mainly by macrophages and microglia. These cells can polarize along a continuum from pro-inflammatory (*classic*, M1) to anti-inflammatory (*alternative*, M2) phenotype. Pro-inflammatory macrophages and microglia release pro-inflammatory cytokines, cytotoxic mediators, and express phagocytic receptors that exacerbate the inflammatory response and promote the clearance of the cell debris 12. Although these factors aim at clearing cell debris, they also produce damage to healthy neighboring cells, exacerbating tissue damage and neurological deficits after SCI 3. In contrast, anti-inflammatory macrophages and microglia release anti-inflammatory cytokines and other mediators that stimulate tissue healing, repair, and recovery of tissue homeostasis 12.

Macrophages and microglia adopt, predominantly, a pro-inflammatory phenotype for the first four weeks after SCI, whereas the presence of those with anti-inflammatory state are succinct 20,34,39. This is related, in part, to the injury environment of the CNS since anti-inflammatory macrophages switch to pro-inflammatory state when transplanted into the lesioned injured spinal cord 34. Understanding the mechanisms that drive macrophages and microglia towards pro-inflammatory and anti-inflammatory phenotypes may be of high relevance for the development of novel therapeutics approached to treat SCI.

Several events have been shown to favor the pro-inflammatory phenotype of microglia and macrophages after SCI, such as TNFα release, iron uptake, and several components of the extracellular matrix and its degradation components 39,40. We previously demonstrated that the insufficient levels of M2 inducers, such as IL-4, in the injured spinal cord was also responsible to hamper the switch of microglia and macrophages towards the anti-inflammatory phenotype 20. Here, we found that the levels of IL-13 were poorly induced in the spinal cord parenchyma after contusion lesion. This cytokine has been extensively used *in vitro* to promote anti-inflammatory polarity to microglia and macrophages 11, and thus, the insufficient levels of this cytokine in the spinal cord post-injury milieu might alter the conversion of these cells towards the anti-inflammatory state. In line with this finding, we provide clear evidence that increasing the bioavailability of rIL-13 in the spinal cord markedly increased the percentage of microglia and macrophages expressing the anti-inflammatory markers Arg1 and CD206. Indeed, the effects of rIL-13 on microglia and macrophages phenotype were very similar to those exerted by rIL-4. We also found that rIL-13, similar to IL-4, induced the appearance of a myeloid cell subset (CD45^high^, CD11b^low^) that are absent in the injured spinal cord after PBS. We previously reported that this myeloid cell population was phenotypically compatible with resolution-phase macrophages 20,41. Resolution-phase macrophages promote clearance of apoptotic cells and trigger the resolution of the inflammatory response leading to tissue homeostasis. Indeed, administration of rIL-4 into the lesioned spinal cord accelerated the clearance of granulocytes from 3 to 6 days post-injury, which is a cardinal feature of enhanced inflammatory resolution 20.

Despite the findings that rIL-4 and rIL-13 led to similar actions on macrophages and microglia in terms of polarizing phenotype and cell counts, only rIL-4 exerted beneficial therapeutic effects after SCI. This is unlikely to be related to the reduced levels of the IL-13 receptor, as rIL-13 successfully promoted anti-inflammatory polarization of microglia and macrophages when injected at 48 h after lesion. This is further supported by the fact that administration of rIL-13 at 18 h post-injury, when levels of 13Rα1 peaked in macrophages and microglia, also failed to show therapeutic efficacy. These findings indicate that rIL-4 and rIL-13 induce differential responses in microglia and macrophages that cannot be clearly appreciable based on the expression of Arg1 or CD206. Moreover, it also highlights that the presence of anti-inflammatory markers in macrophages and microglia does not necessarily imply that they are conducive for tissue repair. This is important to note as this is a widely used to assume the protective response of microglia and macrophages after CNS injury.

To unravel the differential changes exerted by rIL-4 and rIl-13 that could explain their divergent effects on functional recovery after SCI, we performed RNA-seq analysis in macrophages and microglia sorted from the injured spinal cord after injection of PBS, rIL-4 or rIL-13. To our knowledge, this is the first time that transcriptomic profiles of macrophages and microglia have been studied separately in the context of SCI. This analysis revealed that microglia and macrophages have different gene signature after SCI as have been shown previously under physiological conditions 42,43. This suggests that macrophages and microglia have distinct roles in the post-injury milieu. We also found that, from all the active genes detected in macrophages and microglia, some of those differentially modulated by rIL-4 and rIL-13 were associated to cell metabolism. In particular, RNA-seq analysis suggested that rIL-4 fosters genes related to oxidative metabolism as compared to rIL-13.

Previous studies revealed that macrophage activation involves a switch from oxidative phosphorylation to glycolysis in a phenomenon known as Warburg effect 44. Pro-inflammatory macrophages, which are key players of the first line of defense, obtain energy through glycolysis 45. In glycolytic metabolism, glucose uptake and conversion of pyruvate to lactate are increased, and parallelly, respiratory chain activity is depressed leading to ROS production. Parallelly, the pentose phosphate pathway is also induced generating NADPH oxidase which is important for nitric oxide synthesis 46. Although glycolysis is less efficient than mitochondrial respiration in the ATP generation, it provides fast energy necessary for the first stages of the inflammatory response such as proliferation, migration, cytokine synthesis, and phagocytosis 45,47. Energy for these processes is predominately obtained from fatty acids and glucose. In contrast, anti-inflammatory macrophages, which are involved in longer-term functions, such as tissue repair and wound healing, require more efficient metabolic pathways like oxidative phosphorylation 45. After adopting anti-inflammatory polarization, macrophages induce the expression of constituents of the electron transport chain for the oxidative phosphorylation and drive pyruvate into the Krebs cycle. The pentose phosphate pathway is also more limited in anti-inflammatory macrophages 45. These above mentioned changes in macrophage metabolism by the actions of pro-inflammatory and anti-inflammatory cytokines has been also reported in primary microglia cultures 48,49.

In line with RNA-seq analysis, metabolic assays confirmed that rIL-4 enhanced oxidative phosphorylation in myeloid cells in the injured spinal cord as compared to rIL-13 and PBS. Note that rIL-13 also tended to increase mitochondrial respiration although to a much lower extent than rIL-4. This distinctive effect between rIL-13 and rIL-4 on myeloid cell metabolism in SCI may be crucial for explaining the divergent therapeutic responses exerted by rIL-4 and IL-13. Indeed, we found that either activation of macrophages towards the pro-inflammatory state by LPS+INFγ, which is known to induce predominant glycolytic metabolism 45, and chemical inactivation of oxidative phosphorylation in macrophages exerted cytotoxic actions on motoneurons. Anti-inflammatory macrophages stimulated with IL-4 or IL-13 has predominantly oxidative metabolism 45 and did not exert motoneuron death. However, these anti-inflammatory macrophages became cytotoxic and exerted similar detrimental actions in motoneurons when oxidative phosphorylation was inhibited. These data provide clear evidence the mitochondrial respiration in anti-inflammatory macrophages is required for minimizing the harmful responses of this immune cell type. This reinforce our findings that the stimulation of oxidative phosphorylation in myeloid cells by rIL-4 in SCI, but not by IL-13, may be critical for promoting repair after SCI. The mechanisms that prevent the metabolic shift of microglia and macrophages by rIL-13 after SCI are unknown. However, this is likely related to the post-injury milieu since this study, together with previous works, reveals that rIL-13 favors oxidative metabolism in cell culture conditions but not after SCI. This suggests that in the injured spinal cord there are some factors that limit microglia and macrophages from acquiring mitochondrial respiration after rIL-13 administration, in a similar way they prevent the conversion of myeloid cells towards the pro-repair phenotype after lesion 20,34,39. Although the factors that hamper this metabolic shift induced by rIL-13 in myeloid cells in the lesioned CNS are unknown, we speculate that they could be molecules involved in inflammatory response, since some inflammatory mediators such as IFNγ, TNFα, and TLR agonist like LPS, among others, are known to favor glycolytic metabolism in microglia and macrophages.

We also found that rIL-4 and rIL-13 led to different effects in the expression of genes related to DAM. Genes from this signature are considered a risk factor for neurodegenerative diseases as they appear in microglia from Alzheimer's disease, amyotrophic lateral sclerosis, and multiple sclerosis 35. We found that damaged-associated genes were highly expressed in microglia after SCI and that they were markedly modulated by rIL-4. Indeed, all the genes analyzed were significantly downregulated by this cytokine with the exception of *Ccl6*, which was markedly upregulated. The rest of the genes were also modulated by rIL-13 but to a lower extent than rIL-14. The specific contribution of the genes that compose the damage-associated signature to neurodegeneration has not been completely described yet but they seem to be related with phagocytic and lipid metabolic pathways 35. For example, TREM2-APOE pathway is described to be activated in macrophages and microglia after CNS damage and disease and controls the switch from homeostatic to inflammatory conditions by regulating lipid metabolism 36,50,51. Targeting TREM2-APOE signaling eliminated pro-inflammatory macrophages (Ly6C^+^) in Alzheimer's disease mice, restored the homeostatic signature of microglia, reduced inflammation, ameliorated Aβ deposition and Tau pathology, and prevented neuronal death in an acute model of neurodegeneration 36,52. This suggests that TREM2-APOE signaling is detrimental in disease conditions. On the other hand, a recent report also highlighted that Trem2 expression on microglia exerts beneficial actions by promoting myelin debris clearance and remyelination in a model of demyelination 53. Further experiments are needed to elucidate the contribution of DAM to SCI and whether the modulation of DAM signatures by cytokines, such as rIL-4, is important for its beneficial effects.

Overall, the results of the present work elucidate some mechanisms underlying the protective responses of macrophages and microglia after SCI. We highlight that the low levels of M2-inducers, such as IL-4 and IL-13, in the injury milieu prevent microglia and macrophage conversion towards the anti-inflammatory phenotype. Our data demonstrate that the expression of anti-inflammatory markers in microglia and macrophages does not necessarily predict a phenotype conducive for tissue repair. Although this classification is useful when performing *in vitro* studies, this tool may not be relevant *in vivo* due to the complexity of the injury environment. We conclude that other features, such as cell metabolism, should be taken into consideration when studying the beneficial or harmful actions of microglia and macrophages in CNS pathology.

## Supplementary Material

Supplementary figures.

## Abbreviations

A: Antimycin
Arg1: Arginase 1
BMDM: Bone marrow-derived macrophage
BMS: Basso Mouse Scale
CNS: Central nervous system
ECR: Extracellular acidification rate
iNOS: Inducible nitric oxide synthase
JAKs: Janus Kinases
KEGG: Kyoto Encyclopedia of Genes and Genomes
LFB: Luxol Fast Blue
O: Oligomycin
OCR: Oxygen consumption rate
PBS: Phosphate buffer saline
PI3Ks: Phosphoinositide 3 kinases
PCA: Principal Component Analysis
PKB: Protein kinase B
qPCR: Quantitative PCR
rIL-4: Recombinant IL-4
rIL-13: Recombinant IL-13
STAT: Signal transducer and activator of transcription
