# Supplementary material for: Interleukin-4 and interleukin-13 induce different metabolic profiles in microglia and macrophages that relate with divergent outcomes after spinal cord injury
Source: Theranostics. 2021 Oct 3;11(20):9805–20. doi: 10.7150/thno.65203 (PMC8581417; doi:10.7150/thno.65203)
Supplement: Supplementary file 1 — Supplementary figures. [file thnov11p9805s1.pdf]

Supplemental Information

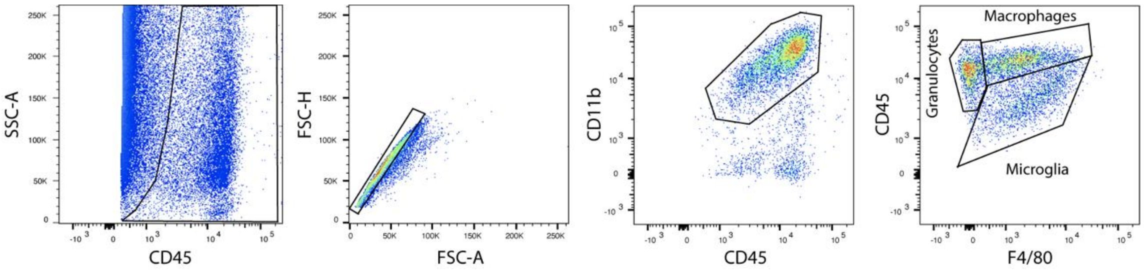

Fig. S1 Representative FACS plots showing the gating strategy to identify granulocytes, macrophages, and microglia in the injured spinal cord.

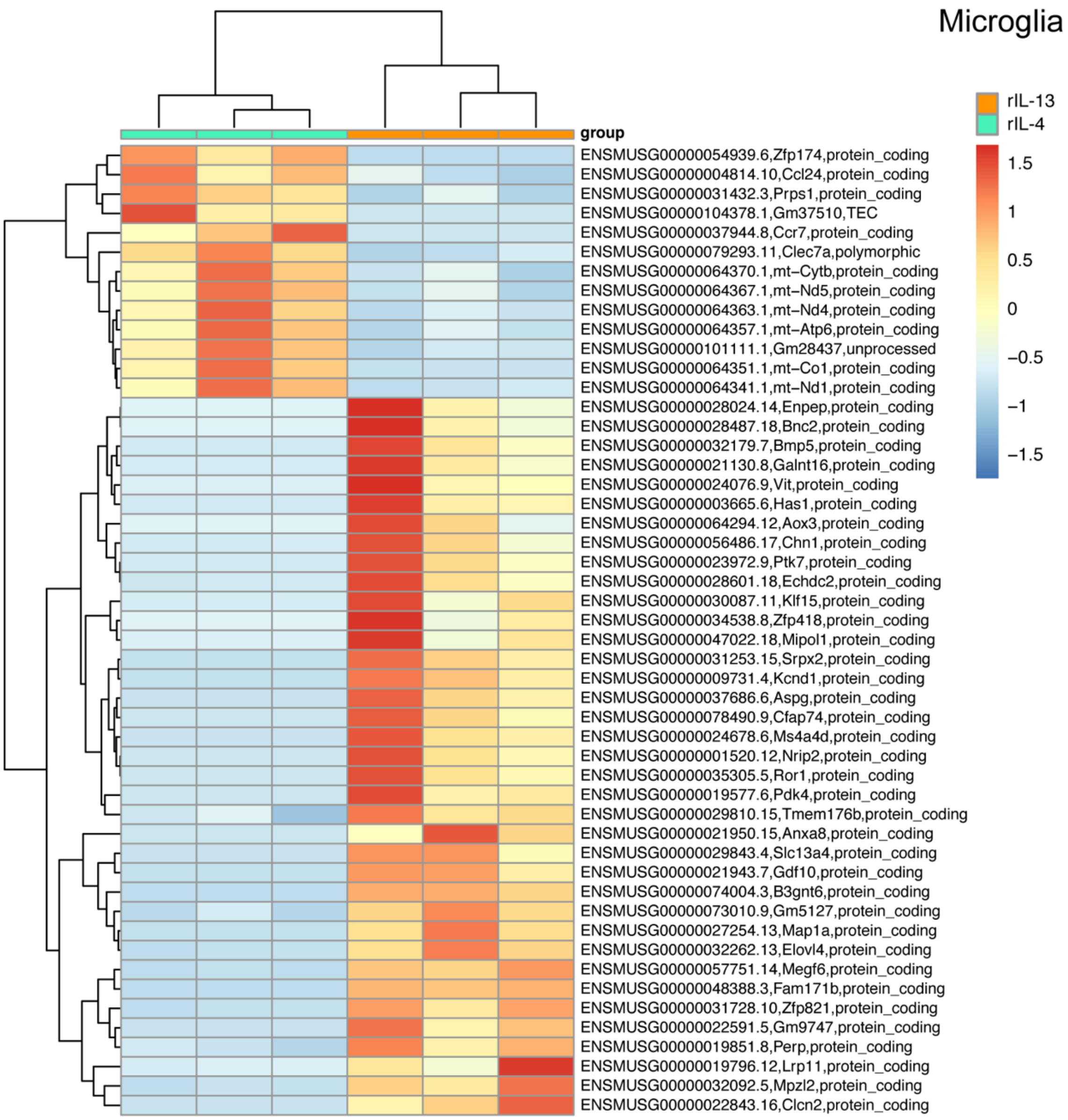

Fig. S2 Heatmap showing the top 50 DE (adjusted p-value < 0.05) genes between rIL-13 and rIL-4 in microglia sorted from the injured spinal cord. Genes upregulated are marked in warm colors whereas genes downregulated are marked in cold colors.

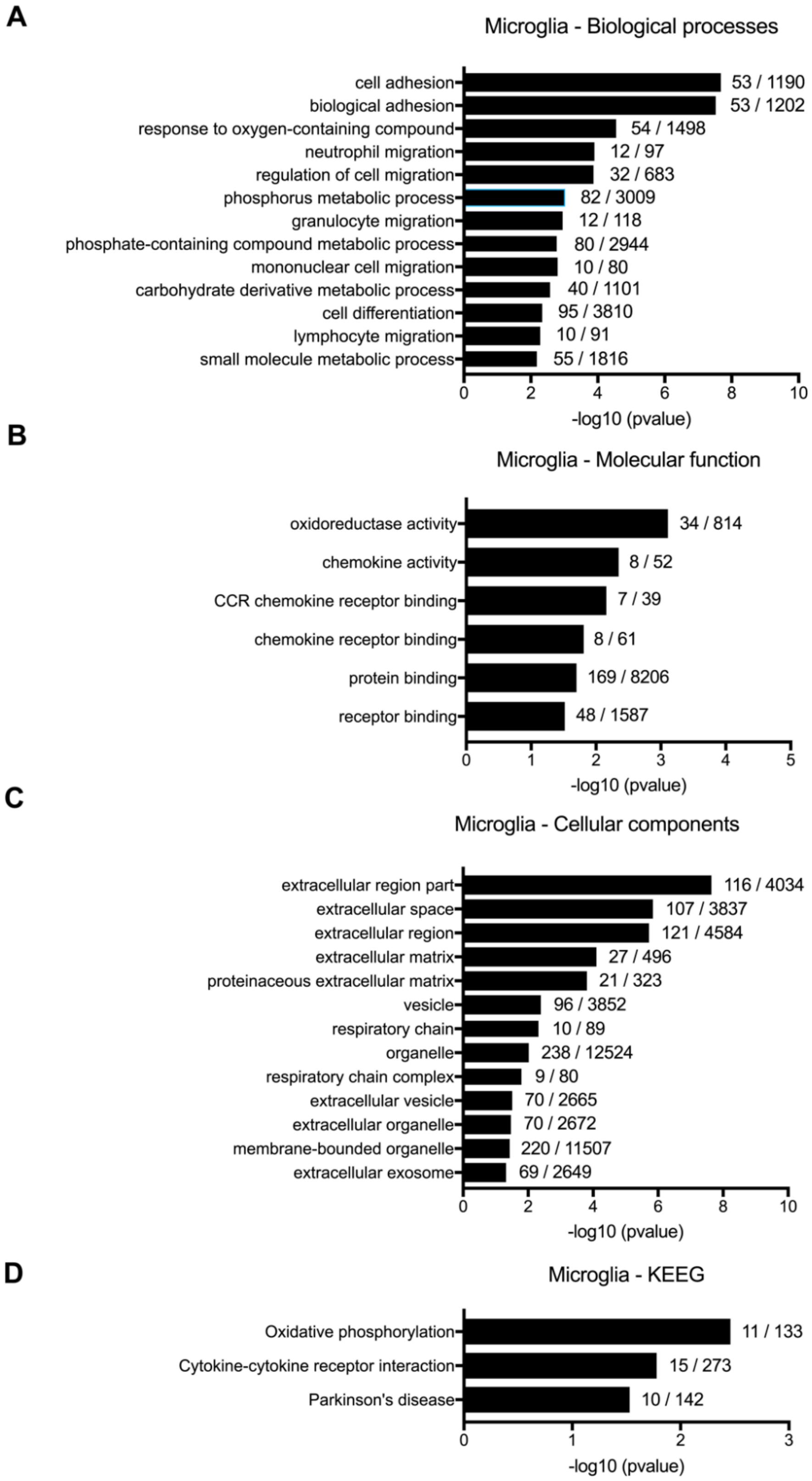

Fig. S3 Selection of pathways significantly altered between rIL-13 and rIL-4 in spinal cord injury sorted microglia. Main biological processes (A), molecular function (B), cellular components (C), and KEGG (D) expressed as −log10 (p-value) are represented. Note −log10 (0.05) = 1.3. Numbers of DE genes respect to the total number of genes assigned to each pathway are indicated.

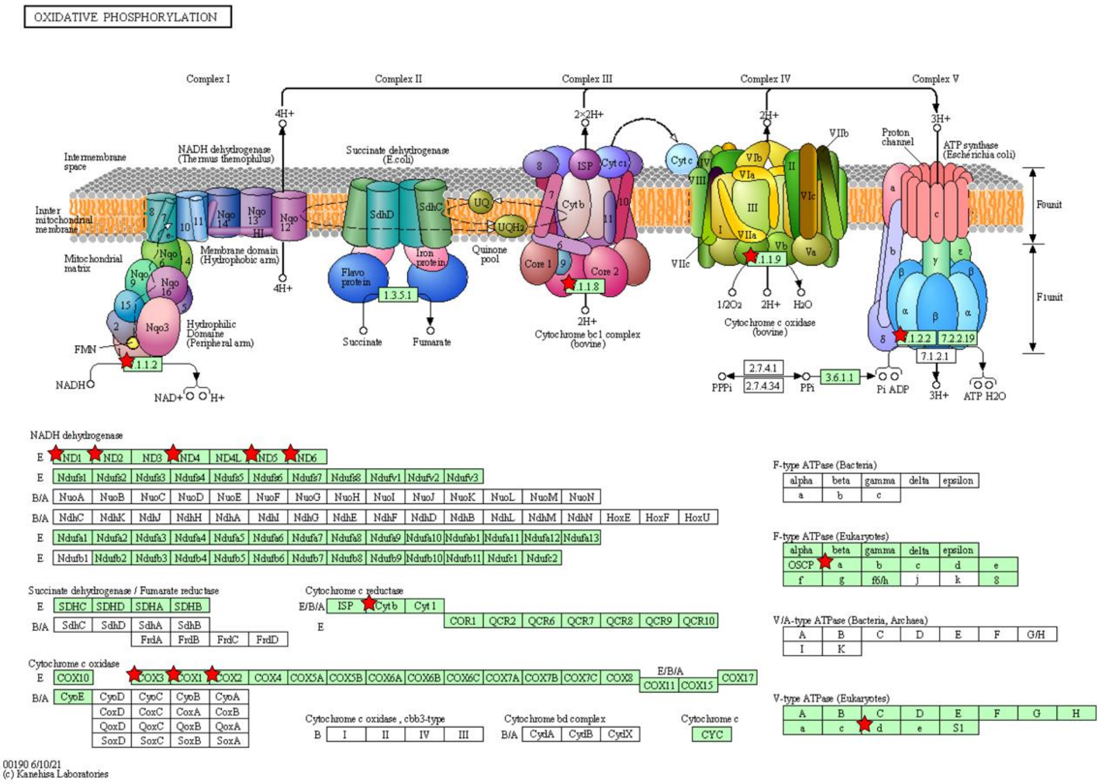

Fig. S4 Oxidative phosphorylation KEGG pathway. Genes differentially expressed in spinal cord injury microglia after rIL-4 and rIL-13 treatment are marked with a red star.

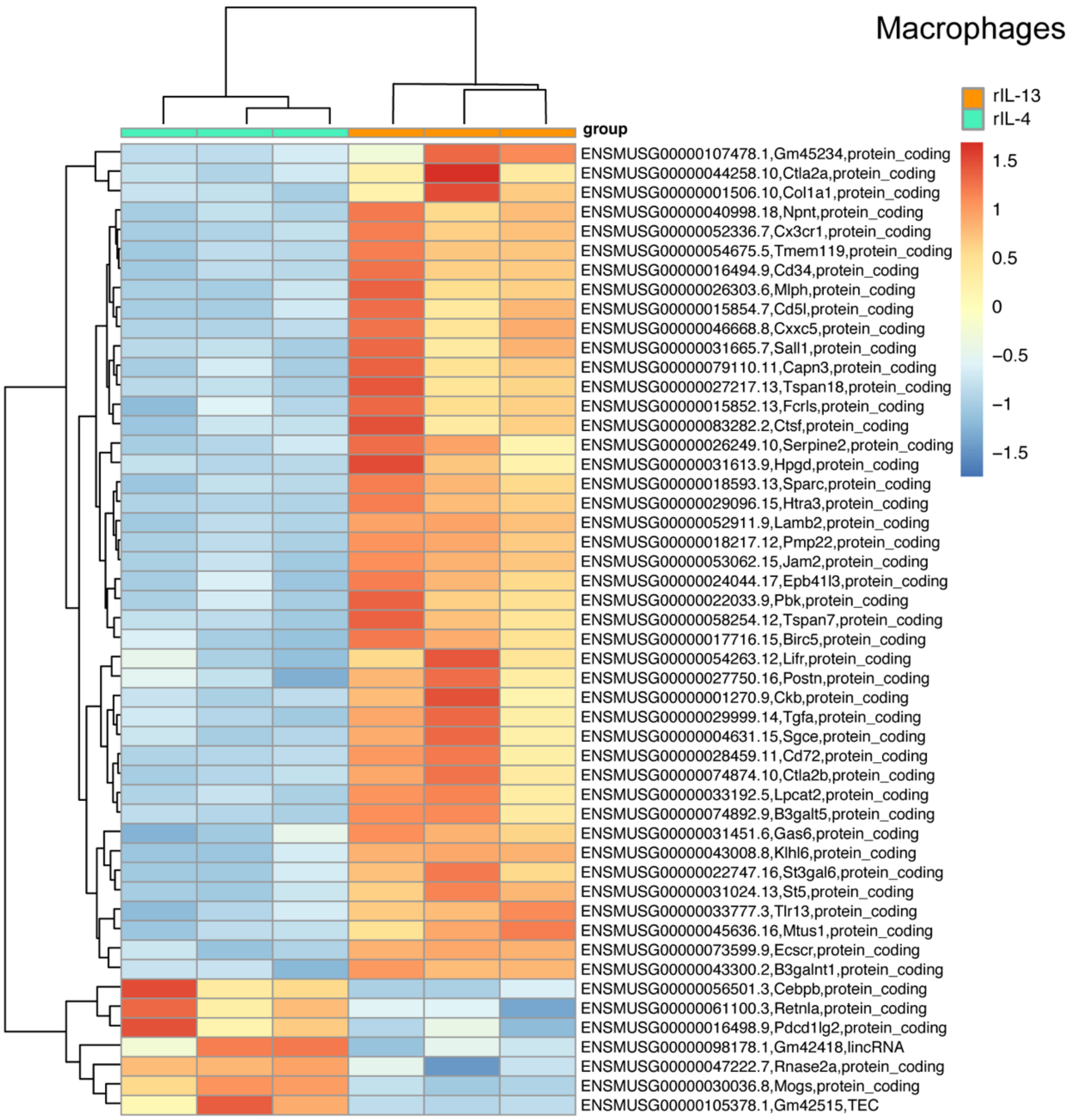

Fig. S5 Heatmap showing the top 50 DE (adjusted p-value < 0.05) genes between rIL-13 and rIL-4 in macrophages sorted from the injured spinal cord. Genes upregulated are marked in warm colors whereas genes downregulated are marked in cold colors.

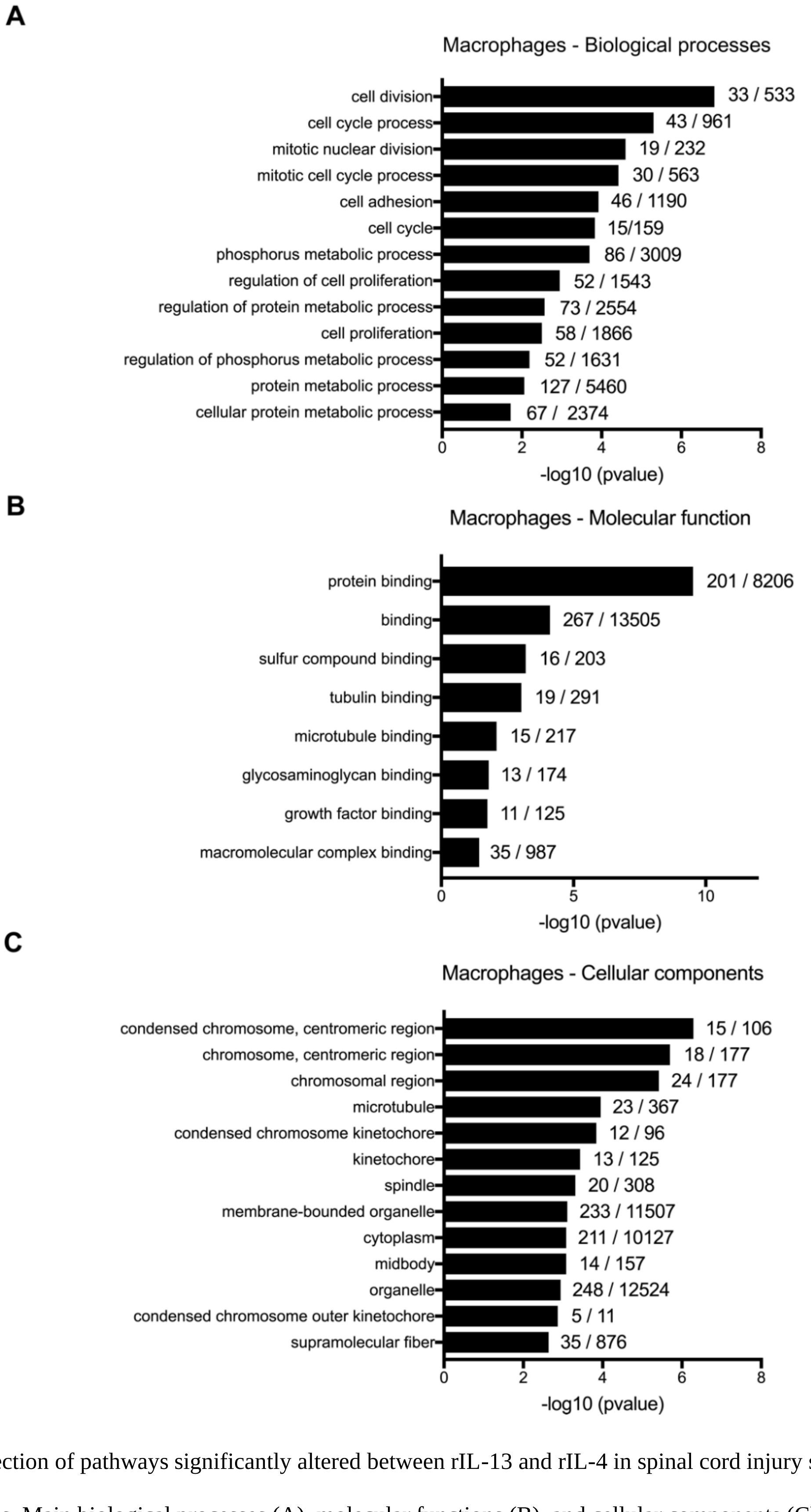

Fig. S6 Selection of pathways significantly altered between rIL-13 and rIL-4 in spinal cord injury sorted macrophages. Main biological processes (A), molecular functions (B), and cellular components (C) expressed as −log10 (p-value) are represented. Note −log10 (0.05) = 1.3. Numbers of DE genes respect to the total number of genes assigned to each pathway are indicated.

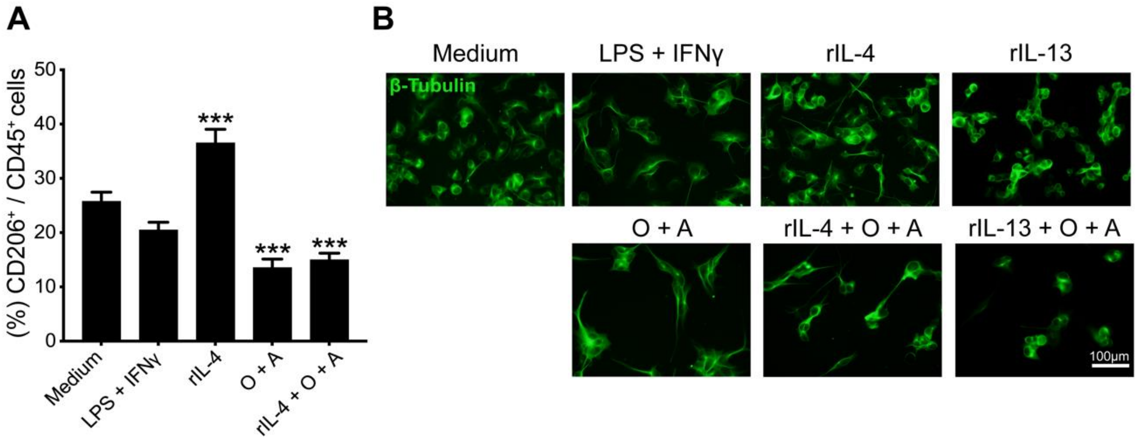

Fig. S7 Inhibition of oxidative phosphorylation on immune cells has a negative impact on neuron survival. (A) Effects of different stimulants on the polarity of BMDMs based on CD206 marker. Oligomycin and antimycin (O+A) were used to inhibit oxidative phosphorylation. (B) Representative fluorescent micrographs of cells showing β-tubulin 3 staining after exposition to different BMDM conditioning mediums. Scale bar 100 µm.
